# Percutaneous triamcinolone injection for upper eyelid retraction in thyroid eye disease

**DOI:** 10.3389/fopht.2024.1388197

**Published:** 2024-05-17

**Authors:** Shaun R. Parsons, Ario Wilson-Pogmore, Timothy J. Sullivan

**Affiliations:** ^1^ Division of Oculoplastic and Orbital Surgery, Royal Brisbane and Women’s Hospital, Brisbane, QLD, Australia; ^2^ Department of Ophthalmology, Queensland Children’s Hospital, Brisbane, QLD, Australia; ^3^ Faculty of Medicine, University of Queensland, Brisbane, QLD, Australia; ^4^ Faculty of Medicine, Griffith University, Gold Coast, QLD, Australia

**Keywords:** thyroid eye disease (TED), upper eyelid retraction, triamcinolone acetate, quality of life, eyelid, orbit - pathology

## Abstract

**Purpose:**

To evaluate percutaneous triamcinolone (TA) injection efficacy in treating upper eyelid retraction (UER) for Australian thyroid eye disease (TED) patients.

**Methods:**

We conducted a retrospective analysis across 8 years and multiple diverse Australian centres identified UER patients who received TA injections. A single operator administered 40mg/1ml TA through upper eyelid skin. Assessments at 4-6 weeks and subsequent eyelid measurements gauged treatment response and complications.

**Results:**

24 patients and 25 eyelids were included in the study. 91.6% were female, mean age 40.8 ± 10.3 years with mean follow-up of 17.5 months (± 18.5). Pre-treatment MRD1 was 6.2mm ± 1.4, and we observed a mean improvement of 2.2mm from pre-treatment to post-treatment (p<0.001). The mean UER measurement before treatment (defined as MRD1 - 4.0mm) was 3.0mm ± 1.3 (range, 0-6mm). After treatment, the mean UER measurement was -0.1mm. Quality of life (QOL) assessment improved significantly, from pre-treatment score of 4.13 ± 2.4 to post-treatment 8.0 ±1.7 (p<0.001).

**Conclusions:**

Percutaneous injection of TA is an effective and safe treatment option for UER in patients with TED. This technique can be performed without upper eyelid eversion, which makes it more tolerable for patients and less complex for the operator compared to the transconjunctival injection approach. Our results show a significant improvement in MRD1 and UER, as well as patient QOL. Moreover, we found a low rate of complications (4.2% induced ptosis) and no cases of raised intraocular pressure. Percutaneous TA injection can greatly reduce the need for eyelid lowering surgery in this patient population.

## Introduction

Upper eyelid retraction (UER) is proposed as the most common clinical feature and a primary diagnostic criterion for thyroid eye disease (TED) (1). While the definition of UER varies across studies (1, 2), it is observed in up to 90% of patients with TED (2–4).

UER can cause lagophthalmos, exposure keratopathy, and negatively affect the quality of life (QOL) due to the psychosocial impact of altered physical appearance (3, 5, 6). The exact pathogenesis of UER remains unclear, with hypothesized mechanisms including levator palpebrae superioris (LPS) muscle fibre enlargement, LPS muscle contraction or fibrosis, increased sympathetic tone in Müller’s muscle, or fixation duress resulting from restriction of the inferior rectus muscle, leading to increased tone of the superior rectus and LPS (7, 8).

Traditional management of UER typically involves symptomatic care while observing for spontaneous improvement and deferring surgical intervention, if necessary, to the quiescent phase (6, 9, 10), which may take 12-24 months or longer. However, patients may prefer an alternative, less invasive, and interim treatment option that provides immediate benefits. In recent times, minimally invasive treatments have gained acceptance due to their availability for intervention during the active phase, faster onset, and favourable safety profiles (11, 12). These include hyaluronic acid (HA) fillers, neuromodulators such as botulinum toxin type A (BTA), and triamcinolone (TA) injections (11).

Upper eyelid injection of TA is a relatively new treatment for UER, with some reports showing promising results via transconjunctival injection for reducing UER (6, 13–18). However, the transconjunctival approach has potential complications, including deep superior sulcus defect, high skin crease, and most commonly, intraocular pressure (IOP) elevation, which has a prevalence range of 4-20% (6, 13–16). Additionally, the transconjunctival approach can be technically more challenging and potentially uncomfortable for the patient, as the upper eyelid is often everted for the procedure (14–17). Percutaneous injection of TA is poorly documented in the literature, with two limited series, including a proof-of-concept report by one of the present authors (19, 20).

The primary objective of this study was to determine the efficacy of percutaneous injection of TA for the treatment of UER in TED in an Australian population. The secondary outcomes included changes in QOL, complication rates, and patient demographic features. The results of this study will guide treatment decisions and contribute to the body of evidence in UER in TED.

## Materials and methods

### Patients

The authors conducted a retrospective, multi-centre, interventional case series between September 1, 2014, and October 1, 2022. Patients were recruited from three distinct sites in Brisbane, Australia, comprising a tertiary institution and two private ophthalmic practices. This multi-centre approach, despite being managed by a single operator, was chosen to ensure broad socio-economic representation among participants. All patients provided consent for treatment and clinical photography, which were taken pre- and post-treatment and appropriately archived by the authors. Following treatment, patients were reviewed at one month to assess the need for re-treatment. If no further treatment was required, patients were followed up at 3, 6, 9 months, and 1 year.

Inclusion criteria were patients with TED and visible UER, with or without signs of active inflammation, who received at least one percutaneous injection of TA for the treatment of UER. Specifically, UER was determined as an upper eyelid position greater than 4.0mm above the margin reflex distance 1 (MRD1), which equates to 4.5mm or more. Patients were included regardless of their previous systemic immunosuppressive therapy, including completed courses of intravenous methylprednisolone (IVMP) and oral prednisolone, and regardless of their current position in the TED disease course. Orbital imaging was assessed to select patients with predominantly thickening of the LPS complex rather than inferior rectus related fibrotic retraction.

Patients who presented with active dysthyroid optic neuropathy (DON) requiring urgent systemic treatment and those who had undergone previous upper eyelid surgery were excluded. None of the patients were receiving concurrent systemic intravenous (IV) steroids.

### Technique

Patients were seated with their heads comfortably supported in a semi-reclined position. The eyelids and surrounding areas were prepped with 5% povidone-iodine using a sterile technique. TA 40mg/mL (Kenacort®-A 40 Aspen Pharmacare Australia) was drawn into a 3mL luer-lock syringe attached to a 25-gauge 1½” (0.5mm x 38mm) needle. To facilitate lower globe displacement, a tongue depressor or the flat handle of Adson forceps was utilized while the patient was instructed to gaze downward.

**Figure 1:**
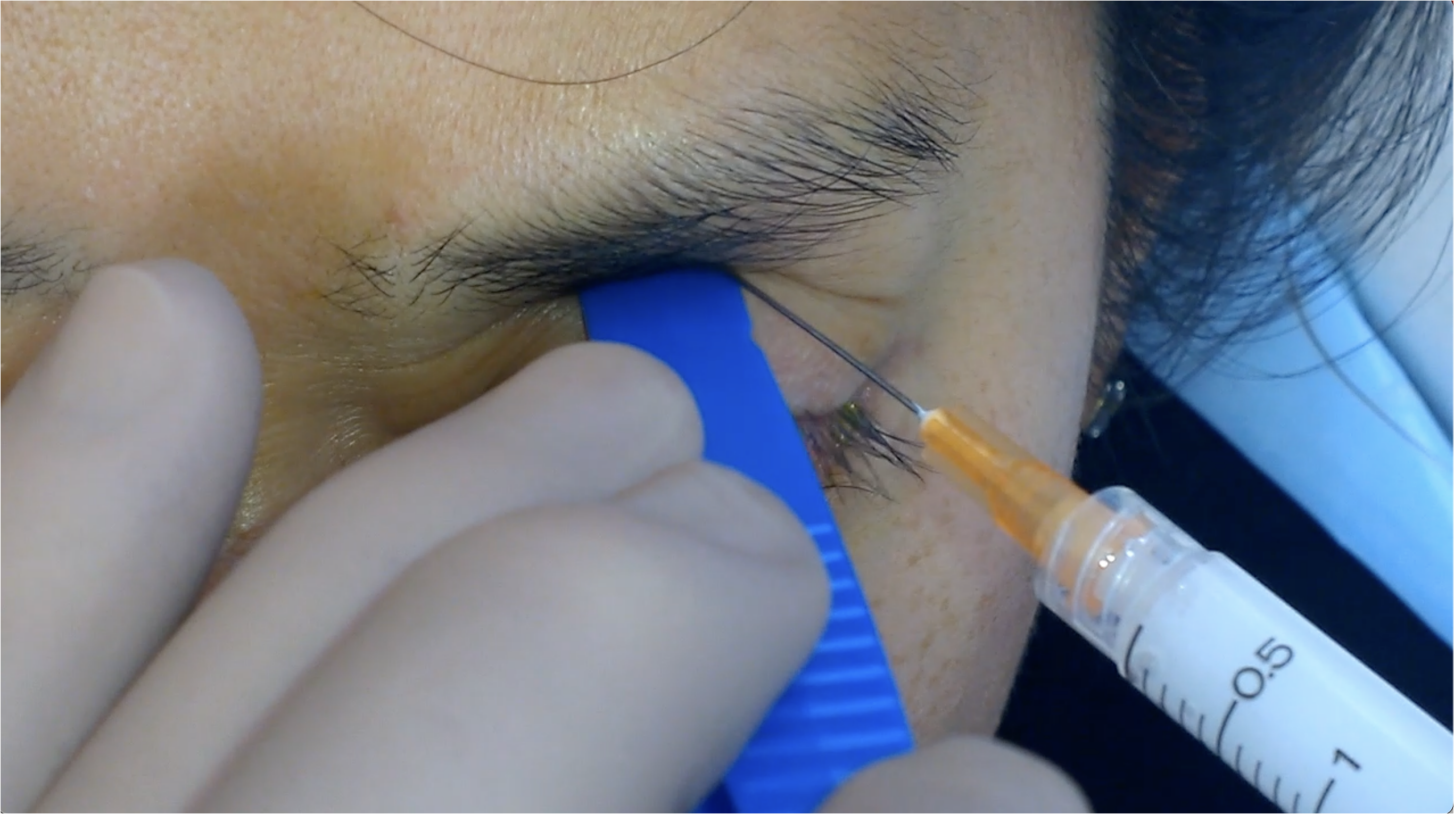
Upper eyelid percutaneous injection technique.

The needle entry point was determined at the skin of the upper eyelid at the junction of the lateral third and medial two-thirds, ensuring placement lateral to the LPS muscle belly. The needle was inserted percutaneously with a slight medial angulation and directed toward the orbital roof with a depth of insertion of approximately 20mm (**Figure 1**).

Aspiration was performed for 3-5 seconds to confirm a lack of blood return, reducing the risk of intravascular injection. Upon safe aspiration, a slow injection of 1ml TA was administered. After injection, a sterile eye pad was applied with gentle pressure and the patient was advised to remain seated with their head upright for 15 minutes.

Following pad removal, immediate assessment of visual acuity and a dilated fundal examination were conducted to rule out embolic phenomena. Each patient received a standard dose of 40mg/1ml TA per injection.

In cases of bilateral UER, treatment was staggered, addressing one eyelid per visit to enhance patient comfort and compliance. Subsequent injections were scheduled at intervals of 4-6 weeks based on the assessment of UER resolution, which was judged by a reduction in lid retraction and improvement in subjective symptoms. The treatment series was capped at three injections per eyelid based on iterative assessments of efficacy and safety outcomes. An exception was noted in an early patient who received six injections, leading to the refinement of the treatment protocol to the current maximum.

### Outcomes

The primary outcome measure was MRD1 using clinical examination and correlated by clinical photographs. All patients underwent comprehensive ophthalmic examination at each visit, including visual acuity, IOP, and the standardized Vision, Inflammation, Strabismus, and Appearance (VISA) assessment from the International Thyroid Eye Disease Society (ITEDS) (21). This included a self-reported QOL survey, using a 1-10 scale. Additionally, serum TSH receptor antibody (TRAb) was taken prior to and at the completion of treatment where possible.

For MRD1, treatment success was defined by the normalization of MRD1 to 4mm or less, or by an improvement in MRD1, indicated by a decrease of at least 0.5mm. Treatment failure was identified if there was no reduction in MRD1 or if the MRD1 increased from the baseline measurement. All patients were assessed at each visit for complications, which were recorded.

*Research Ethics.* This study was approved by the Royal Brisbane and Women’s Hospital research ethics committee. This research study adhered to the tenets of the Declaration of Helsinki.

## Results

Our study included 24 patients and 25 eyelids (12 right, 13 left). The majority of the patients (91.6%, n=22) were females, with an average age of 40.8 ± 10.3 years (range: 24-59 years) (**Table 1**). Eighteen patients were of Caucasian background and the remaining 6 were of Asian descent. The mean follow-up period was 17.5 ± 18.5 months. Of the patients, 20.8% (n=5) were current smokers and 70.8% (n=17) were taking selenium. The mean duration of TED symptoms before the first TA injection was 12.9 ± 12.3 months (range: 2-48 months). At the time of inclusion in the study, 58.3% (n=14) were taking carbimazole for Graves’ disease management, 25% (n=6) were receiving thyroxine supplementation following radioactive iodine I-131 (n=5) or thyroidectomy (n=1), and 16.6% (n=4) were not receiving any systemic treatment.

**Table 1:** Patient Demographics.

No. Patients	24
No. Eyelids	25
Gender, n (%)	
Male	2 (8.3%)
Female	22 (91.7%)
Age (y)	40.8 ± 10.3 years
Current smoker, n (%)	5 (20.8%)
Selenium Supp. n (%)	17 (70.8%)
Injections mean ± std	2.1 ± 1.0
Onset symptoms to first injection	12.9 ± 12.3

Regarding the number of injections, one patient received injections in both eyelids, and the mean number of injections per patient was 2.1 ± 1.0 (range: 1-6). One injection was needed for 20.8% (n=5) of the patients, two injections for 58.3% (n=14), three injections for 16.7% (n=4), and six injections for 4.2% (n=1).

**Figure 2:**
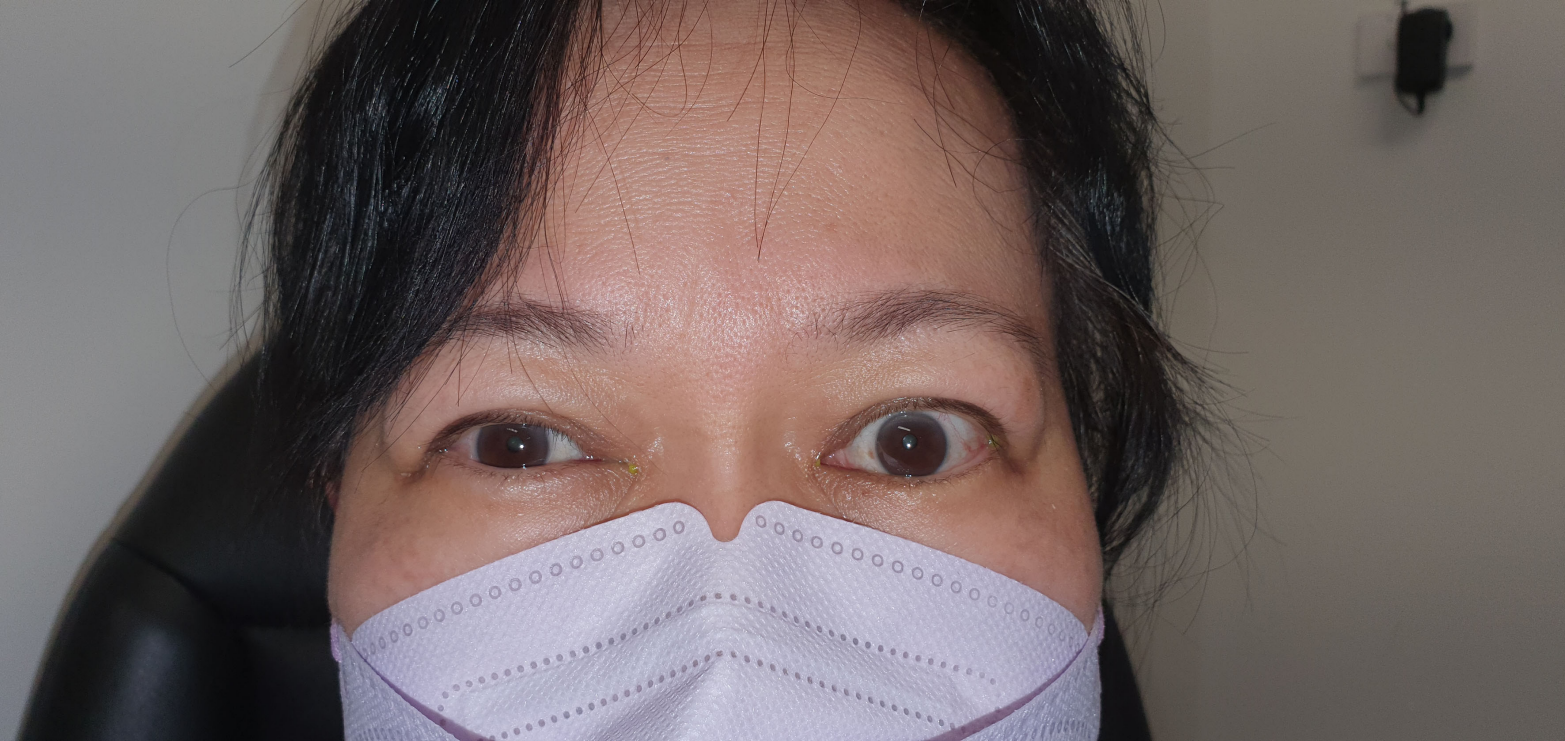
Pretreatment of left upper eyelid retraction.

**Figure 3:**
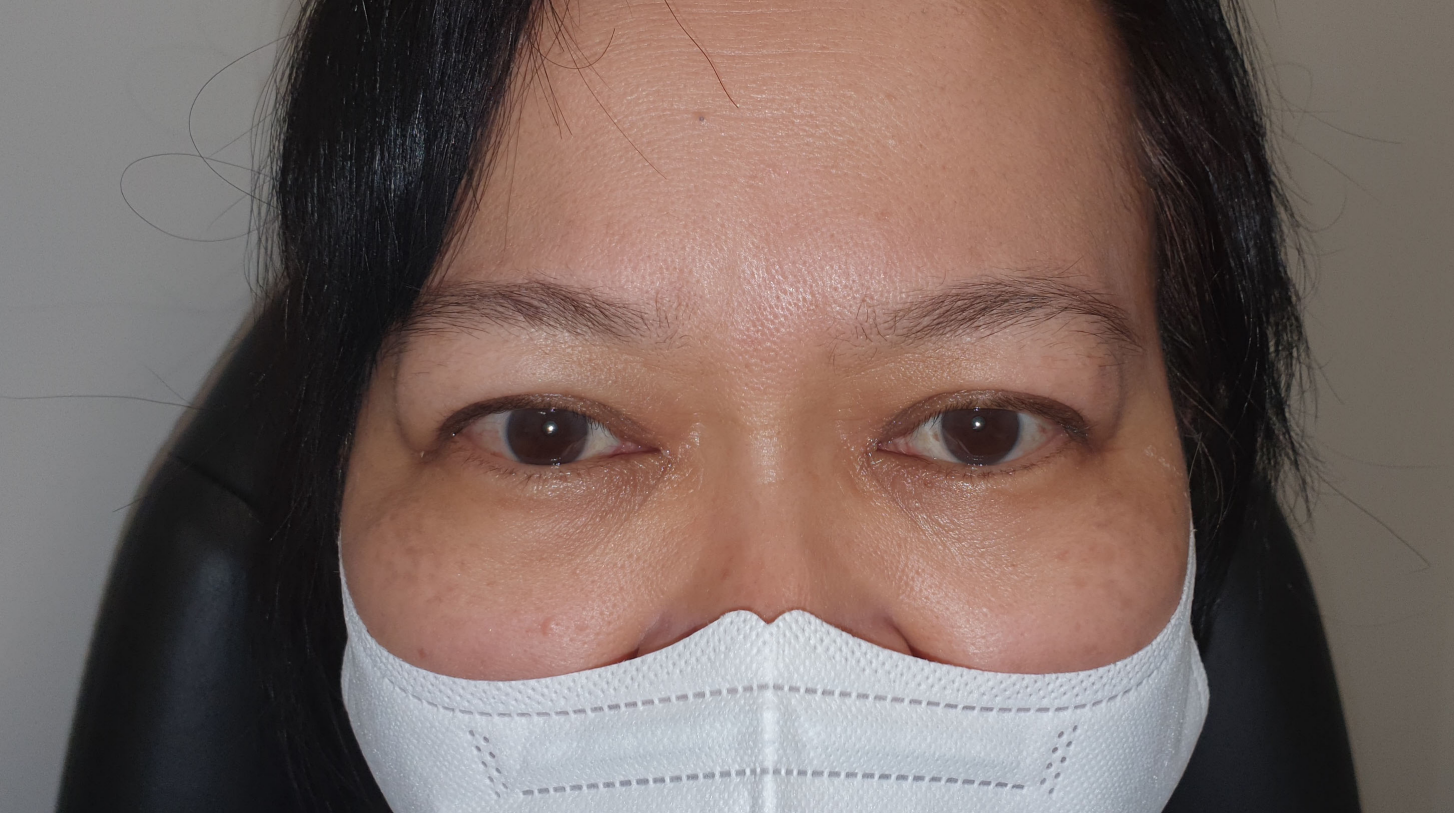
Post treatment of left upper eyelid retraction.

Before treatment, the mean MRD1 was 6.2mm ± 1.4 (range: 4.5-10mm) (**Figure 2**). The mean improvement in MRD1 was 2.2mm from pre- to post-treatment (p<0.001). The mean pre-treatment UER was 3.0mm ± 1.3 (range: 0.5-6mm), which decreased to a mean post-treatment UER of -0.1mm (**Table 2**, **Figure 3**).

**Table 2:** Clinical features Pre-Treatment and Post-Treatment treatment.

	Pre	Post	Change	Correlation	Sig
MRD1	6.2	3.98	2.2	0.642	<0.001
UER	2.24	-0.18	2.42	0.668	<0.001
QOL	4.13	8.02	3.89	0.388	<0.001

MRD1 Margin Reflex distance 1.

UER Upper Eyelid Retraction.

QOL Quality of Life.

In terms of QOL assessment, there was a significant improvement from a pre-treatment mean of 4.13 ± 2.4 to 8.0 ± 1.7 (p<0.001).

Pre-treatment TRAb was available for 86% (n=21) of patients, and post-treatment for 79.2% (n=17). The mean TRAb decreased from 8.53 ± 8.95 IU/L (range: 0.23-31) pre-treatment to 1.63 ± 1.70 IU/L (range: 0.3-7) post-treatment. Paired-sample t testing revealed a mean difference of 8.79 ± 9.21 IU/L following treatment (p=0.002).

**Figure 4:**
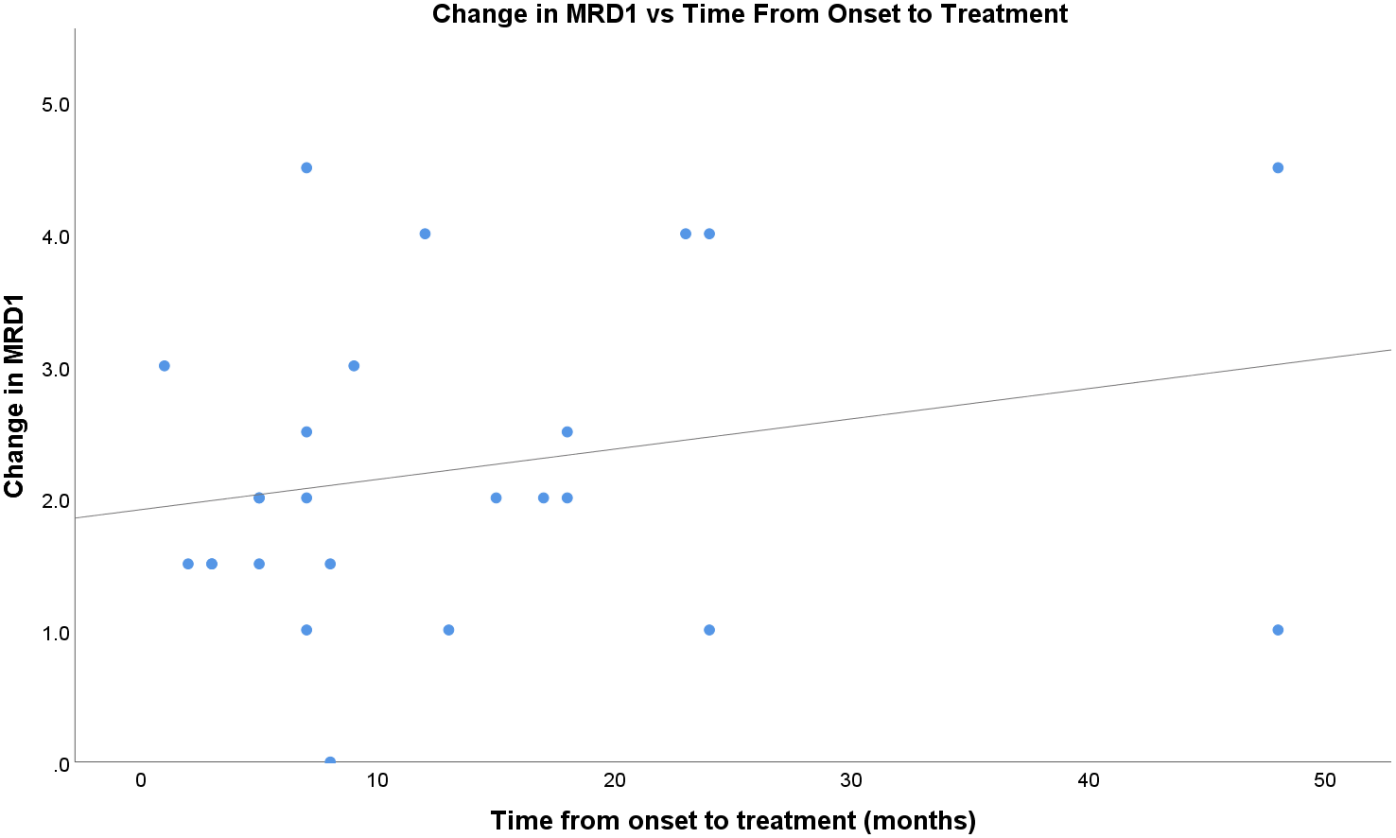
Change in MRD1 vs time, in months, from onset of TED to first percuatenous upper eyelid injection.

The time from onset of TED symptoms to the first injection did not appear to correlate well with the treatment response (**Figure 4**). In this sample, patients who received treatment later in their clinical course generally showed a positive trend in the treatment response compared to those in the acute initial phase. The fitted regression model indicated that the duration of symptoms was not a significant predictor of treatment response (r=0.24; p=0.26).

As for complications, no patients experienced clinically significant IOP elevation greater than 20mmHg, and thus, no patient required IOP-lowering topical treatment. One patient underwent single eyelid ptosis surgery, and another underwent a single eyelid lowering procedure (blepharotomy) due to an incomplete response after receiving a single TA treatment.

## Discussion

The study results indicate that percutaneous TA injection effectively treated UER. The mean improvement of UER was 2.42mm, resulting in a mean post-treatment UER of -0.18mm, which was statistically significant. These findings are consistent with other studies that used the transconjunctival injection technique, which reported mean improvements in MRD1 of 2.1mm by Young et al. (6), 2.19mm by Xu et al. (16) and 0.6-1.1mm by Lee et al. (14) at the final follow-up. The natural history of UER retraction in TED has been observed, showing a reduction from a mean of 6.1mm at initial presentation to 4.3mm after 48 months without any intervention (8). The results of this study suggest that our described intervention can achieve a lower final UER more quickly than what is typically expected based on natural history.

Moreover, the QOL reported score within the VISA assessment improved from 4.13 pre-treatment to 8.02, which was statistically significant. To our knowledge, this is the only study to demonstrate significant improvement in QOL in TED-related UER with localized eyelid injection treatment. UER has been reported as the most common sign of TED and may result in exposure keratopathy and negative psychosocial implications (1, 2, 5). Previously, managing UER was challenging and unrewarding for patients and clinicians, with limited options. Surgical upper eyelid lowering procedures are generally delayed for the inactive phase of the disease and can be invasive, irreversible, and relatively unpredictable, with a recognized risk of over or under correction (6, 17). Alternative injections, including BTA and more recently HA gel fillers, have been proposed as treatment options however the effects may be temporary or unpredictable with time (15, 22–24). TED compromises patients’ quality of life, with the majority reporting limitations in daily activities and reduced self-confidence due to changes in their appearance (25). The clinical severity of the disease correlates with decreased QOL and UER can lead to significant psychosocial problems (5, 25). This study’s findings support the use of TA injections as an effective treatment for enhancing QOL in patients with TED related UER.

The measured TRAb levels reduced from a mean of 10.43 IU/L pre-treatment to 1.64 IU/L post-treatment, which was statistically significant. Second and third generation TRAb assays have demonstrated >97% sensitivity and specificity in the diagnosis of Graves’ Disease (GD) (26). Additionally, serum TRAb levels have been demonstrated to directly correlate to TED clinical activity score (CAS) (27). Previous studies have generally demonstrated a decline in TRAb levels over months to years with both medical and surgical treatment of GD (28, 29). Our results suggest that our studied cohort had active disease at the initiation of treatment, and following treatment, the serum TRAb levels were significantly reduced soon after the perilevator TA injection. Although it is difficult to draw meaningful conclusions relating to the effect of TA injections and serum TRAb levels given variability in systemic hyperthyroid treatment and timing of measurement, intraorbital steroids may play a role in reducing TRAb levels more quickly than the natural history. Future studies with a control group would be beneficial to provide more conclusive evidence of causality of this observed association.

Our study included patients with UER regardless of their position on the disease course and previous immunosuppressive treatments. Previous studies have shown a greater effect of TA injection for UER in early active disease. Improvement was demonstrated in 86.3% of the active group compared with 25% of the inactive group by Lee et al. (14). Young et al. (6) had greater treatment success in the active group (92.5%), but there was substantial success in the inactive group (72.0%). Our study found a slightly greater effect in patients who received treatment longer after the onset of symptoms compared with early treatment (**Figure 4**). The proposed mechanism of TA in TED-related UER is multifactorial and includes an anti-inflammatory effect on the levator and Müller’s muscles, steroid-induced ptosis, degenerative changes in the levator muscle and/or Müller’s muscle. The use of orbital imaging is a well-established for diagnosing TED, but the range of eyelid symptoms is not explained through thickening of the LPS alone (30, 31). Research using magnetic resonance imaging (MRI) to assess LPS changes before and after transconjunctival triamcinolone acetonide (TA) injections for UER found that LPS thickness decreased regardless of the treatment’s effectiveness (30). Even with this reduction in LPS thickness, the group that did not respond to treatment exhibited fibrotic complications in Müller’s muscle and LPS, and adhesions in the surrounding tissue, which led to ongoing UER. This suggests that the efficacy of TA injections may also involve reducing fibrotic changes, beyond simply decreasing LPS thickness.

One patient (4.2%) experienced a complication of induced ptosis following two TA injections, with their MRD1 decreasing from 6mm pre-treatment to 2mm post-treatment. This patient underwent successful levator advancement surgery. Induced ptosis has been reported in a similar prevalence following transconjunctival TA (6, 15). One patient (4.2%) underwent upper eyelid lowering procedure (blepharotomy). This patient elected to receive only one TA injection which reduced their MRD1 from 9mm to 8mm and subsequently decided to undergo blepharotomy procedure, rather than further TA injections. There were no cases of raised IOP >20mmHg at the one-month follow-up visit or any subsequent visits, and no patients required IOP-lowering therapy during follow-up. A recent study of orbital TA injections also found no cases of IOP rise (32) whilst previous studies on transconjunctival injection of TA have reported a higher IOP response rate, ranging from 4-20% (6, 13–16). Some of these patients have required IOP-lowering medication for up to 12 months, but there are no reports of permanent glaucomatous damage or the need for glaucoma-filtration surgery.

There are some limitations to our study that should be considered. Firstly, the design was retrospective and lacked a placebo control group. We were able to compare our results with a natural history study on UER in TED for comparison. There can be expected improvement in UER with the natural course of the disease that was not accounted for. This improvement typically occurs over 18-24 months. Additionally, treatment frequency and schedule varied based on patient response and preference, although this reflects real-world experience. Defining an optimal frequency and interval for TA injections would be beneficial. Additionally, this treatment may not be effective for all pathophysiological variations of TED-related UER. Specifically, fibrotic retraction related to the inferior rectus muscle, which was not assessed in this study.

In conclusion, percutaneous TA injection is an effective and safe treatment option for TED-related UER, which improves patient QOL. Our study demonstrated that this treatment can be effective in both early active disease and later presentations during the inactive phase. It may be considered a simpler and more comfortable alternative to transconjunctival injection, as it does not require upper eyelid eversion. Additionally, we did not encounter the complication of raised IOP, and there was a reduced need for surgical eyelid lowering procedures.

## Data availability statement

The original contributions presented in the study are included in the article/supplementary material. Further inquiries can be directed to the corresponding author.

## Ethics statement

The studies involving humans were approved by Royal Brisbane and Women’s Hospital research ethics committee. The studies were conducted in accordance with the local legislation and institutional requirements. The participants provided their written informed consent to participate in this study. Written informed consent was obtained from the individual(s) for the publication of any potentially identifiable images or data included in this article.

## Author contributions

SP: Conceptualization, Data curation, Formal analysis, Investigation, Methodology, Writing – original draft, Writing – review & editing. AW-P: Data curation, Formal analysis, Methodology, Writing – original draft, Writing – review & editing. TS: Conceptualization, Data curation, Investigation, Methodology, Supervision, Writing – original draft, Writing – review & editing.
